# Ruthenium Compounds Differentially Inhibit Group IA and IIA Snake Venom Phospholipase A_2_ Anticoagulant Activity

**DOI:** 10.3390/ijms27073228

**Published:** 2026-04-02

**Authors:** Vance G. Nielsen, Sarah A. Nielsen

**Affiliations:** 1 Department of Anesthesiology, College of Medicine, University of Arizona, Tucson, AZ 85750, USA; 2 Department of Ecology and Evolutionary Biology, College of Science, University of Arizona, Tucson, AZ 85724, USA; sarahn2@arizona.edu

**Keywords:** snake venom, neurotoxicity, anticoagulation, factor Xa, ruthenium, thrombelastography, snake venom enzyme inhibitors

## Abstract

Neurotoxicity caused by snake venom phospholipase A_2_ (PLA_2_) activity derived from coral snakes (e.g., *Micrurus tener*, *Micrurus fulvius*, group IA PLA_2_) and some rattlesnakes (e.g., *Crotalus scutulatus*, group IIA PLA_2_) is medically significant. Of interest, the catalytic site of PLA_2_ also binds to activated clotting factor X, causing anticoagulation. Given that ruthenium (Ru)-containing compounds have been demonstrated to inactivate hemotoxic venoms in a solvent-dependent manner (e.g., 0.9% NaCl, phosphate-buffered saline), we wished to determine if RuCl_3_ would cause solvent-dependent inhibition of snake venom group IA and group IIA PLA_2_ in human plasma with thrombelastography. It was determined that RuCl_3_ significantly decreased the anticoagulant effects of group IA PLA_2_ derived from *M. tener* and *M. fulvius* venoms in the presence of 0.9% NaCl, but not phosphate-buffered saline. In contrast, group IIA PLA_2_ anticoagulant activity derived from *C. scutulatus* venom was inhibited by RuCl_3_ in both solvents. It is concluded that the different ions formed by RuCl_3_ in different solvents may interact with novel disulfide bridges unique to group IA and IIA PLA_2_ or through some other mechanism. In vivo validation of Ru-based enzyme inhibitor effects on neurotoxicity associated with either group IA or IIA remains a critical translational issue.

## 1. Introduction

Neurotoxic envenomation by coral snakes (e.g., *Micrurus tener*, *Micrurus fulvius*) or certain rattlesnakes (e.g., *Crotalus scutulatus*, venom type A) results in apneic death of prey animals or regional/systemic weakness of larger animals, such as humans and companion animals [1]. Phospholipase A_2_ (PLA_2_) is one of the primary components of these snake venoms that inflicts neurotoxicity, intravascular hemolysis, and death [1,2,3,4,5,6,7,8]. PLA_2_ comprises esterolytic enzymes that cleave the 2-acyl linkage of phosphoglycerides [9,10,11,12,13,14,15,16,17], with the superfamily of PLA_2_ composed of 15 different groups [9,10,11,12,13,14,15,16,17]. With regard to venoms containing PLA_2_, group IA PLA_2_ is found in Elapidae venoms [10,11,14,15,17], groups IIA and IIB PLA_2_ are contained in Crotalidae and Viperidae venoms, respectively [10,11,14,15,17], and group III PLA_2_ is found in Apidae and Helodermatidae venoms [10,11,14,15]. While the amino acid sequence of the catalytic site of PLA_2_ is highly conserved throughout the groups (e.g., most containing a critical histidine^48^ residue), there are a great many differences between groups IA, IIA, and III [9,10,11,12,13,14,15,16,17,18,19,20,21]. Specifically, group IA PLA_2_ has a molecular weight of 13–15 kD, contains seven disulfide bridges, has a unique disulfide bridge between amino acids 11 and 77, and tends to comprise calcium-dependent enzymes [10,11,14,17]. Further, group IIA PLA_2_ has a molecular weight of 13–15 kD, contains seven disulfide bridges, has a unique disulfide bridge between amino acids 50 and 137, and is typically calcium-independent for activity [10,11,14,17]. Lastly, group III PLA_2_ has a molecular weight of 15–18 kD, contains eight disulfide bonds, and usually requires calcium for activity [10,11,14,17,18,19,20,21]. Taken as a whole, PLA_2_, as contained in diverse sources of venom, is complex.

In addition to neuromuscular paralysis, PLA_2_ can cause a variety of other phenomena: vascular injury that causes regional edema and hemorrhage [22], pain [23,24,25,26,27], and inhibition of coagulation in vitro [28,29,30]. Of these, anticoagulation is a process caused by non-enzymatic binding of PLA_2_ to the coagulation enzyme activated factor X (FXa), inhibiting prothrombinase activity and the generation of thrombin [28,29,30,31]. Critically, while the bonding of PLA_2_ to FXa is a mechanism of anticoagulation, the catalytic site histidine^48^ residue is required for such bonding [30,31]. This single-amino-acid nexus of anticoagulation via nonenzymatic binding and enzymatic-based injury allows for a unique assessment of a variety of novel antitoxins without the need for in vivo models. The low-molecular-weight compound varespladib is a potent inhibitor of PLA_2_ that binds to its histidine^48^ [32], and it has been demonstrated to significantly attenuate snake and bee venom-mediated tissue injury in vivo [33,34,35,36,37,38,39,40,41]. Varespladib also inhibited snake venom group IA and IIA PLA_2_-mediated anticoagulation [42,43,44], and it is notably derived from *M. tener* and *M. fulvius* [43]. Another low-molecular-weight molecule, ursolic acid, was found to bind to group IA and IIA PLA_2_ derived from snake venom and to decrease in vitro hemolysis and murine paw edema [45]; further, ursolic acid inhibited group IIA PLA_2_-mediated anticoagulation from venom derived from *C. scutulatus* [46]. Considered altogether, the literature supports the concept that the in vivo efficacy of antivenoms that are based on binding to PLA_2_ could be assessed by the in vitro inhibition of PLA_2_ anticoagulation—especially with respect to antivenoms containing low-molecular-weight molecules that bind to histidine.

One such genus of site-directed, precision enzyme inhibitors includes solutions that contain ruthenium (Ru)-containing radicals and ions that have been demonstrated to inhibit PLA_2_ anticoagulation [46,47,48,49]. One such compound that generates a Ru radical species, tricarbonyldichlororuthenium(II) dimer, inhibits the anticoagulant effect of group IA PLA_2_ (*M. fulvius* [48]), group IIA PLA_2_ (*C. scutulatus* [48] and *C. adamanteus* [49]), and group III PLA_2_ (*Apis mellifera* [50]). Ruthenium chloride (RuCl_3_) is another compound demonstrated to inhibit group IIA PLA_2_ [47], metalloproteinase [51], and serine protease-mediated [52] effects on coagulation. Critically, the tricarbonyldichlororuthenium(II) dimer (hereafter referred to as carbon monoxide releasing molecule-2, CORM-2) has been demonstrated to exert its inhibitory effects on PLA_2_ activity in a histidine-quenchable manner [50]. Other Ru(III)-containing compounds have been found to bind to histidine in serum proteins [53], and both Ru(II)-containing [54,55] and Ru(III)-containing [56] compounds have been found to break or become incorporated in disulfide bonds. When combined with the preceding data concerning PLA_2_ molecular structure, it is possible that there is more than one potential binding site (e.g., catalytic site histidine^48^, disulfide bridges) available for different Ru-containing molecules to interact with groups IA, IIA, and III PLA_2_. Further, the efficacy of RuCl_3_ as an enzymatic inhibitor depends on the solution it is dissolved in and the venom protein assessed; specifically, rattlesnake metalloproteinase activity is inhibited by RuCl_3_ dissolved in 0.9% NaCl or phosphate-buffered saline (PBS), whereas a serine protease containing *Bothrops* venom is only inhibited by RuCl_3_ dissolved in 0.9% NaCl (hereafter normal saline, NS) [52]. Thus, it would be of great interest to investigate if PLA_2_ activity in the medically important venoms is inhibited by different RuCl_3_-derived ions.

**Figure 1:**
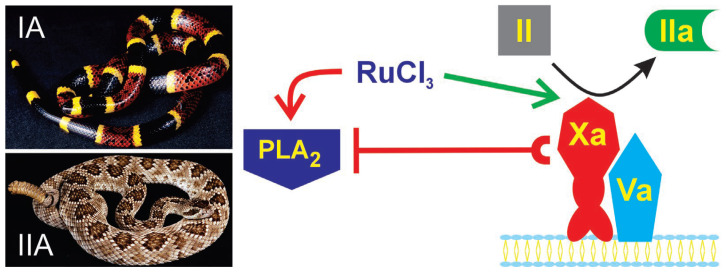
Schemes of prothrombinase enhanced activation by RuCl_3_ in PBS or PLA_2_-mediated inhibition. Prothrombinase, composed of a complex of membrane-bound activated FX (Xa) and activated Factor V (Va), converts prothrombin (II) to thrombin (IIa) to subsequently cause plasmatic coagulation. Ions derived from RuCl_3_ dissolved in PBS enhance prothrombinase activity (green arrow). PLA_2_ derived from *M. tener* venom (IA group) and *C. scutulatus* Type A venom (IIA group) inhibits prothrombinase activity by binding to Xa in a nonenzymatic manner (red line with a C). Lastly, ions formed from RuCl_3_ dissolved in PBS and perhaps NS may irreversibly bond to group IA and IIA PLA_2_, preventing PLA_2_-mediated inhibition of prothrombinase activity.

However, before such experimentation is undertaken, it must be noted that Ru-containing compounds can modulate plasmatic coagulation. Specifically, CORM-2 enhances coagulation by binding to fibrinogen, improving it as a substrate for thrombin [57]. Further, RuCl_3_ dissolved in PBS enhances prothrombin activity, resulting in decreased time to onset of coagulation and increased velocity of clot growth but no change in clot strength [58]. Thus, RuCl_3_ under the correct conditions can enhance the prothrombinase-mediated generation of thrombin in plasma, and group IA and IIA PLA_2_, in turn, can bind to FXa and prevent thrombin generation. These relationships of enhanced prothrombinase activation or inhibition are depicted in Figure 1.

Considered as a whole, the aforementioned information supports the possibility that ions derived from RuCl_3_ may be effective or ineffective in inhibiting group IA and IIA snake venom PLA_2_ activity. While a site-directed, Ru-based enzyme inhibitor composed of a mixture of CORM-2 and RuCl_3_ dissolved in PBS has been effective in inhibiting hemotoxic venoms in vivo [59], perhaps using NS as the solvent would provide superior neutralization with different venoms, as was the case when testing a *Bothrops* venom in vitro [52]. Thus, when designing Ru-based enzyme inhibitors with the goal of inhibiting neurotoxic snake venoms containing PLA_2_, it would be critical to know which solvent would be ideal to use with RuCl_3_ to inhibit group IA and IIA PLA_2_ activity.

To fill these important gaps of knowledge concerning RuCl_3_-based enzyme inhibitor effects on group IA and IIA PLA_2_ activities, the following goals were formulated. First, the effects of the low concentrations of RuCl_3_ (dissolved in PBS or NS) remaining following isolated exposure to venom on the coagulation kinetics of the test plasma sample were to be defined. Second, isolated exposure of *M. tener* and *M. fulvius* venom (group IA PLA_2_) to RuCl_3_ dissolved in NS or PBS was to be performed to determine if there were differences in inhibition of PLA_2_ anticoagulant activity in human plasma. Lastly, isolated exposure of *C. scutulatus* venom (group IIA PLA_2_) to RuCl_3_ dissolved in NS or PBS was to be achieved to determine if there were any differences in inhibition of PLA_2_ anticoagulant activity in plasma.

## 2. Results

### 2.1. Effects of RuCl_3_ Dissolved in NS or PBS on Plasmatic Coagulation Kinetics

**Figure 2:**
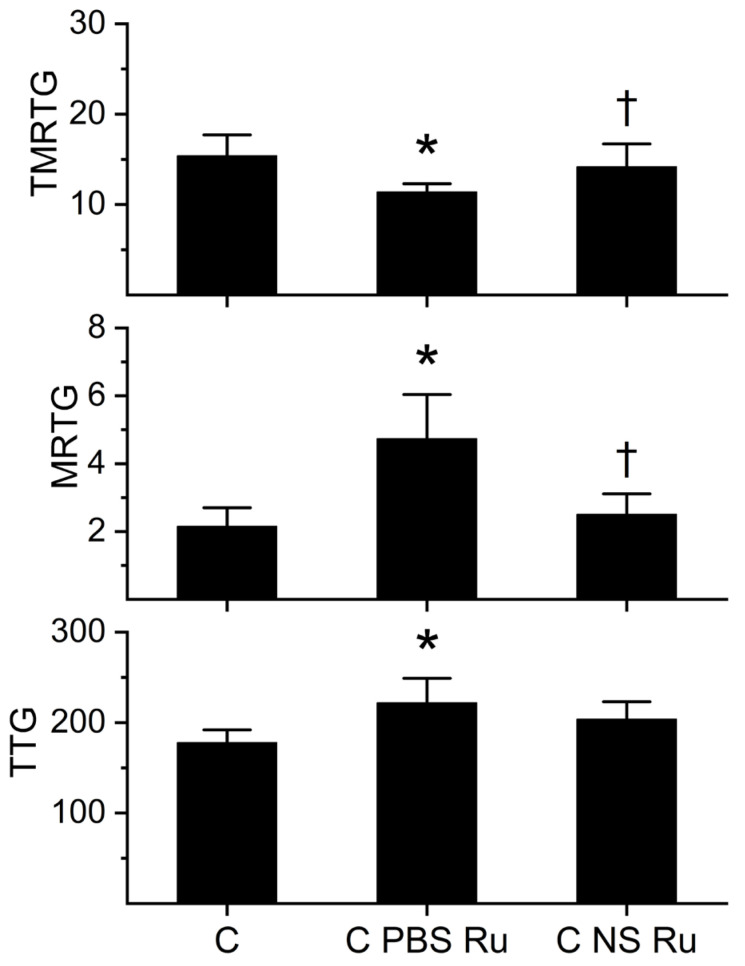
Effects of RuCl_3_ dissolved in PBS or NS on human plasmatic coagulation kinetics. C = control (1% addition NaCl); C PBS Ru = 1 µM RuCl_3_ dissolved in PBS; C NS Ru = 1 µM RuCl_3_ dissolved in NS. All conditions: n = 6 replicates. TMRTG = Time to maximum rate of thrombus generation (minutes), a measure of the speed of onset of coagulation; MRTG = maximum rate of thrombus generation (dynes/cm^2^/second), a determination of the velocity of clot formation; TTG = total thrombus generation (dynes/cm^2^), the viscoelastic resistance or clot strength of the thrombus. Data were analyzed with one-way analysis of variance (ANOVA) with a Holm–Sidak post hoc test. * *p* < 0.05 vs. C; ^†^ *p* < 0.05 vs. C PBS Ru.

Given that RuCl_3_ could potentially have an effect on coagulation, a series of experiments was performed, wherein human plasma was exposed to a 1% addition (*v*/*v*) of NS without any RuCl_3_ present (control conditions), RuCl_3_ dissolved in NS, or RuCl_3_ dissolved in PBS. The final concentration of RuCl_3_ was 1 µM, as this would be the concentration observed when venom was exposed to 100 µM RuCl_3_ in isolation prior to being placed into plasma samples as a 1% addition to assess changes in anticoagulant activity. The results of these experiments are displayed in Figure 2. Coagulation kinetics were determined with thrombelastography as previously described, with data collected over a 25-minute period [46,47,48,49,50,51]. RuCl_3_ dissolved in PBS significantly decreased the time to onset of coagulation (TMRTG) and increased the velocity of clot growth (MRTG) in plasma compared to plasma samples exposed to only NS or RuCl_3_ dissolved in NS. There were no significant differences in TMRTG or MRTG between plasma samples exposed to NS or RuCl_3_ dissolved in NS. However, while final thrombus strength (TTG) was significantly greater in samples exposed to RuCl_3_ dissolved in PBS compared to plasma with NS addition, samples exposed to RuCl_3_ dissolved in NS had values not significantly different from the other two conditions. As this last observation supported the possibility of some influence of RuCl_3_ dissolved in NS on coagulation, subsequent experiments compared plasma samples exposed to RuCl_3_, exposed venom, or RuCl_3_ alone with matched solvents.

### 2.2. Effects of RuCl_3_ Dissolved in NS or PBS on the Anticoagulant Activity of M. tener Venom in Plasma

**Figure 3:**
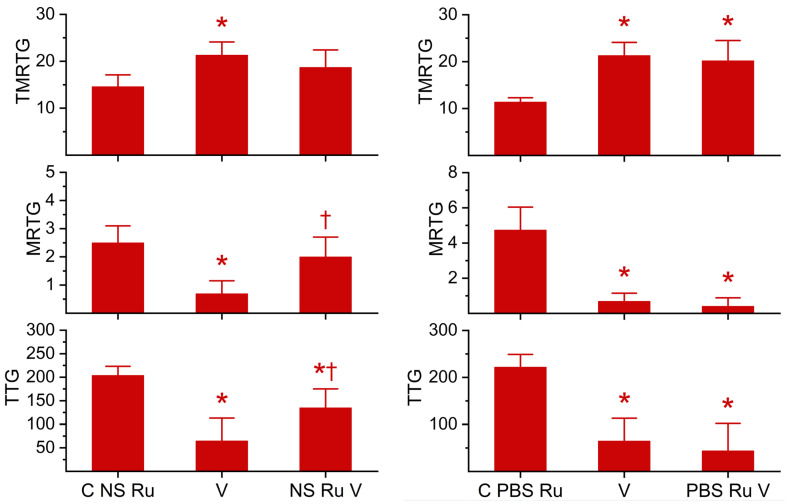
Effects of RuCl_3_ dissolved in NS or PBS on the anticoagulant activity of *M. tener* venom. C NS Ru = 1 µM RuCl_3_ dissolved in NS. C PBS Ru = 1 µM RuCl_3_ dissolved in PBS; V = 100 ng/mL *M. tener* venom in NS; NS Ru V = *M. tener* venom exposed to 100 µM RuCl_3_ dissolved in NS; PBS Ru V = *M. tener* venom exposed to 100 µM RuCl_3_ dissolved in PBS. All conditions n = 6 replicates. Data were analyzed with one-way ANOVA with a Holm–Sidak post hoc test. * *p* < 0.05 vs. C NS Ru or C PBS Ru; ^†^ *p* < 0.05 vs. V.

Based on the methodology presented elsewhere [46,47,48,49,50,51], it was determined that a concentration of 100 ng/mL of *M. tener* venom was sufficient to cause marked anticoagulation as determined by thrombelastography during a 25-minute data collection period. As depicted in both sets of panels in Figure 3, *M. tener* venom significantly increased TMRTG, decreased MRTG, and decreased TTG values compared to control conditions. When exposed in isolation to RuCl_3_ in NS, as seen in the right set of panels, *M. tenor* venom had significantly decreased anticoagulant activity manifested as a TMRTG value not different from the control, MRTG values not different from control values but significantly greater than unexposed venom, and significantly greater TTG values than those observed from venom not exposed to RuCl_3_. In sharp contrast, as observed in the panel on the right side of Figure 3, *M. tener* venom’s anticoagulant activity was not inhibited significantly when exposed to RuCl_3_ in isolation in PBS, with all thrombelastographic parameters differing from control values but not from conditions observed with *M. tener* venom alone. In summary, RuCl_3_ significantly inhibited this venom only in the presence of NS, not PBS.

### 2.3. Effects of RuCl_3_ Dissolved in NS or PBS on the Anticoagulant Activity of M. fulvius Venom in Plasma

**Figure 4:**
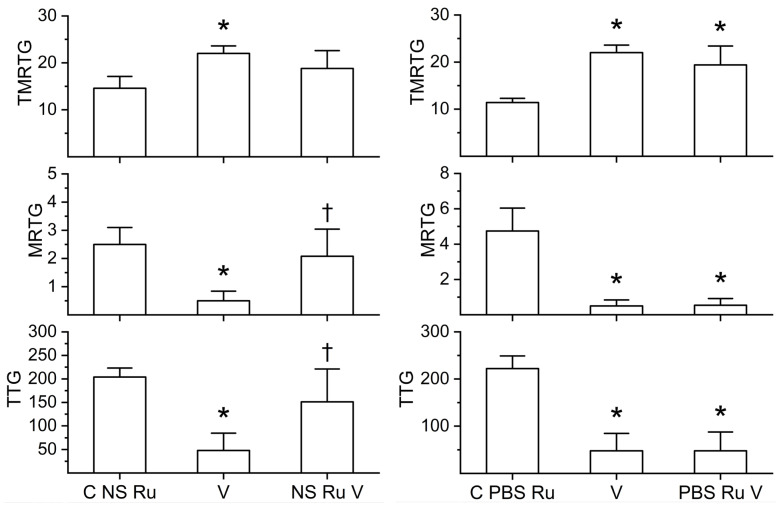
Effects of RuCl_3_ dissolved in NS or PBS on the anticoagulant activity of *M. fulvius* venom. C NS Ru = 1 µM RuCl_3_ dissolved in NS. C PBS Ru = 1 µM RuCl_3_ dissolved in PBS; V = 100 ng/mL *M. tener* venom in NS; NS Ru V = *M. fulvius* venom exposed to 100 µM RuCl_3_ dissolved in NS; PBS Ru V = *M. fulvius* venom exposed to 100 µM RuCl_3_ dissolved in PBS. All conditions: n = 6 replicates. Data were analyzed with one-way ANOVA with a Holm–Sidak post hoc test. * *p* < 0.05 vs. C NS Ru or C PBS Ru; ^†^ *p* < 0.05 vs. V.

As with the previous venom, using methods found elsewhere [46,47,48,49,50,51], it was determined that a concentration of 250 ng/mL of *M. fulvius* venom was sufficient to cause marked anticoagulation as determined by thrombelastography during a 25-minute data collection period. As displayed in both sets of panels in Figure 4, *M. fulvius* venom significantly increased TMRTG, decreased MRTG, and decreased TTG values compared to control conditions. When exposed in isolation to RuCl_3_ in NS, as depicted in the left set of panels, *M. fulvius* venom had significantly decreased anticoagulant activity manifested as a TMRTG value not different from the control, MRTG values not different from control values but significantly greater than unexposed venom, and TTG values not different from control values but significantly greater than those observed with venom not exposed to RuCl_3_. Critically, as observed in the panel on the right side of Figure 3, *M. fulvius* venom anticoagulant activity was not inhibited significantly when exposed to RuCl_3_ in isolation in PBS, with all thrombelastographic parameters different from control values but not different from conditions with *M. fulvius* venom not exposed to RuCl_3_. In summary, as with *M. tenor* venom, RuCl_3_ significantly inhibited *M. fulvius* venom only in the presence of NS, not PBS.

### 2.4. Effects of RuCl_3_ Dissolved in NS or PBS on the Anticoagulant Activity of C. scutulatus Venom in Plasma

**Figure 5:**
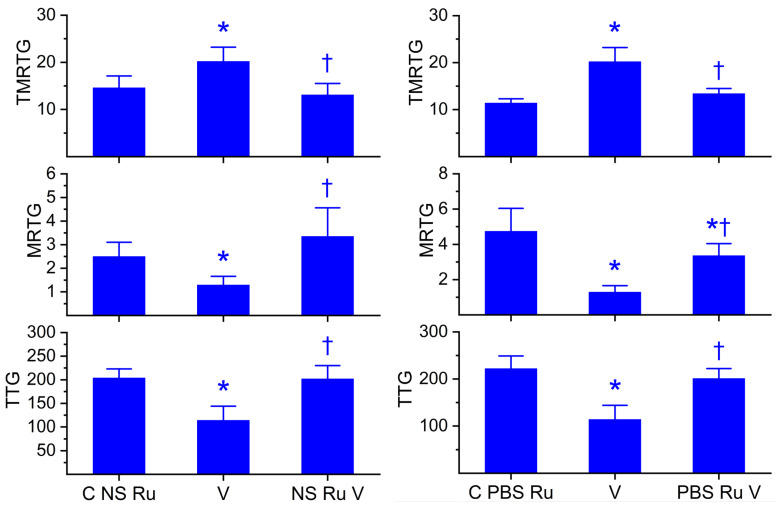
Effects of RuCl_3_ dissolved in NS or PBS on the anticoagulant activity of *C. scutulatus* venom. C NS Ru = 1 µM RuCl_3_ dissolved in NS. C PBS Ru = 1 µM RuCl_3_ dissolved in PBS; V = 100 ng/mL *M. tener* venom in NS; NS Ru V = *C. scutulatus* venom exposed to 100 µM RuCl_3_ dissolved in NS; PBS Ru V = *C. scutulatus* venom exposed to 100 µM RuCl_3_ dissolved in PBS. All conditions: n = 6 replicates. Data were analyzed with one-way ANOVA with a Holm–Sidak post hoc test. * *p* < 0.05 vs. C NS Ru or C PBS Ru; ^†^ *p* < 0.05 vs. V.

Utilizing previously presented methods [46,47,48,49,50,51], it was determined that a concentration of 125 ng/mL of *C. scutulatus* venom was sufficient to cause marked anticoagulation as determined by thrombelastography during a 25-minute data collection period. As displayed in both sets of panels in Figure 5, *C. scutulatus* venom significantly increased TMRTG, decreased MRTG, and decreased TTG values compared to control conditions. When exposed in isolation to RuCl_3_ in NS, as depicted in the left set of panels, *C. scutulatus* venom had significantly decreased anticoagulant activity manifested as a TMRTG value not different from the control, MRTG values not different from control values but significantly greater than unexposed venom, and TTG values not different from control values but significantly greater than those observed with venom not exposed to RuCl_3_. Unlike the preceding two venoms, *C. scutulatus* venom anticoagulant activity was also inhibited by RuCl_3_ in the presence of PBS, as seen in the right set of panels in Figure 5. Isolated exposure of *C. scutulatus* venom in PBS with RuCl_3_ resulted in TMRTG values not different from the control but significantly lower than venom not exposed to RuCl_3_; MRTG values significantly lower than control values but significantly greater than RuCl_3_ naïve venom; and TTG values not different from control values but significantly greater than those observed with venom not exposed to RuCl_3_.

## 3. Discussion

The present investigation achieved its goals of defining the effects of RuCl_3_, dissolved in NS or PBS, on plasmatic coagulation under conditions of limited thrombin generation and on the anticoagulant activity of venoms known to contain group IA or IIA PLA_2_. As RuCl_3_ dissolved in PBS, and to a much lesser extent NS, resulted in procoagulant effects on plasmatic coagulation, control conditions had to be matched to experimental conditions, wherein all samples had a residual concentration of 1 µM RuCl_3_ present. Previous work demonstrated that 50 µM RuCl_3_ enhanced prothrombinase activity in the same limited thrombin-generating plasmatic environment [58], so the finding that only 1 µM RuCl_3_ would still have effects on coagulation was unexpected. With regard to snake venom PLA_2_ anticoagulant activity inhibition by RuCl_3_, there were group-specific differences that were solvent-dependent (e.g., NS vs. PBS). Some potential reasons for these group-specific differences in RuCl_3_-mediated inhibition of PLA_2_ activity will subsequently be presented.

The present investigation has identified important phenomena concerning the inhibition of IA and IIA PLA_2_ by RuCL_3_, but the precise mechanisms responsible remain to be elucidated. As mentioned in the introduction, group IA and IIA PLA_2_ vary by containing unique disulfide bridges and calcium-dependent activity [10,11,14,17]. Further, group IA PLA_2_ usually functions as monomeric molecules, although they can be dimeric depending on the pH of the environment [60,61,62]. The neurotoxic PLA_2_ contained in *C. scutulatus* venom, a group IIA PLA_2_, is a heterodimer with one basic and one acidic molecule [8]. Besides potential differences in potential vulnerabilities to RuCl_3_ secondary to interactions with disulfide bridges, the conformation of PLA_2_ as a monomer or dimer could block or accommodate RuCl_3_-mediated inhibition of anticoagulant activity. In the case of *C. scutulatus* venom, perhaps one PLA_2_ of the heterodimer is vulnerable to RuCl_3_ in NS, while the other is vulnerable to RuCl_3_ in PBS. Further, there may be a role played by the pH of the microenvironment that enhances or inhibits RuCl_3_-mediated PLA_2_ activity inhibition. The pH of NS is 4.5 to 7.0 [63], whereas the PBS used in this investigation is stated to have a pH range of 7.2–7.6 at 25 °C by the manufacturer. It is possible that group IA PLA_2_ is more vulnerable to RuCl_3_-mediated inhibition in the acidic environment of NS, whereas group IIA PLA_2_ may be inhibited by RuCl_3_ in both acidic and normal pH conditions. Also of note, the ions that can form from RuCl_3_ in NS can only involve NaCl (154 mM), whereas in PBS, there are several ions present. In the product used in this investigation, the molar concentrations of the constituents of PBS are as follows: NaCl (137 mM), KCl (2.7 mM), Na_2_HPO_4_ (10 mM), and KH_2_PO_4_ (1.8 mM). All these characteristics of the PLA_2_, NS, and PBS are potentially important for explaining the observed inhibition of PLA_2_, but the aforementioned possible biomolecule–inorganic ion interactions are speculative at this time. There is no mass spectroscopic or other data provided by the present study to provide further mechanistic insight into the manner by which inhibition of PLA_2_ is affected by RuCl_3_. Such endeavors are beyond the scope of the present preliminary study, which included the identification of any differences in the inhibition of group IA and IIA PLA_2_ by RuCl_3_. Only future investigation will determine the precise mechanisms underlying the important data generated by the present work.

Ru-containing radicals and ions have been identified to inhibit aberrations of coagulation caused by hemotoxic and neurotoxic snake venom in vitro [48,49,51,52,64]. Specifically, the procoagulant effects of serine-protease-containing venoms [52,64], anticoagulant effects of metalloproteinase-containing venoms [51,64], and venoms containing different groups of PLA_2_ [47,48,49] have been inhibited by radicals formed from CORM-2 and ions formed from RuCl_3_. Venoms containing procoagulant serine protease activity are inhibited by CORM-2 uniformly [64], but RuCl_3_ seems to inhibit activity in a species-specific manner [52,65]. Specifically, *Calloselasma rhodostoma* venom procoagulant activity is inhibited by RuCl_3_ in PBS [65], whereas *Bothrops moojeni* [65] and *Bothrops diporus* [52] venoms did not have activity inhibited by RuCl_3_ in PBS. However, *Bothrops diporus* venom was inhibited by RuCl_3_ in NS [52]. In the case of venoms with anticoagulant metalloproteinases, CORM-2 and RuCl_3_ dissolved in NS or PBS inhibit activity [51]. As for snake venom group IA or IIA PLA_2_-mediated anticoagulant activity, CORM-2 uniformly inhibits all venoms tested in the cited works [48,49], as well as in other literature not listed. For the convenience of the reader, various snake venom enzymes, Ru-containing compounds, and associated solvents are listed in Table 1, with inhibition or lack of inhibition by the compounds listed with the cited literature.

While the present investigation adds to our knowledge about the conditions necessary for various Ru-containing molecules to inhibit or not inhibit important snake venom enzymes, the efficacy of these inorganic enzyme inhibitors in vivo may be unpredictable. Specifically, when a solution of these molecules is injected into the subcutaneous space containing recently injected venom, the concentrations of anions and cations in vivo will not resemble NS or PBS, and the pH will likely be somewhat acidic and secondary to venom-mediated damage. For example, in a rabbit model, a subcutaneously injected solution of CORM-2 and RuCl_3_ dissolved in PBS inhibited the coagulopathic effects of subcutaneously injected *Calloselasma rhodostoma* venom and *Bothrops moojeni* venom to a similar extent [59]. This in vivo result, equivalent inhibition of serine proteases with CORM-2 and RuCl_3_ dissolved in PBS [59], would not have been predicted based on in vitro results [65]. Nevertheless, perhaps Ru-based enzyme inhibitors should be tailored to the venoms most likely to cause medically important injuries, such as using NS as the carrier solution when treating patients envenomed with group IA PLA_2_-containing venoms. Future preclinical and clinical investigations will be required to translate the findings of the present and previous investigations into potential therapies for venomous snake bites.

The present investigation has limitations. First, the conditions of isolated exposure of the venoms tested in the present study were not physiological secondary to the need to minimize the final concentration of RuCl_3_ in the plasma mixture to minimize the procoagulant effects of RuCl_3_ as a confounding factor. The experimental design of the present investigation was meant to maximize the ability to discern solvent-mediated effects on the enzyme-inhibiting properties of RuCl_3_ on the anticoagulant activity of the venoms tested. The present study was never meant to be a translational work that recreates the subcutaneous tissue of a living system. These in vitro findings, therefore, must be viewed with caution, with future in vivo investigations determining if the same molecular interactions between RuCl_3_- and PLA_2_-abundant venoms persist. While the anticoagulant effects of *C. scutulatus* venom have been demonstrated to be totally attributable to PLA_2_ activity with thrombelastography by exposing the venom to ursolic acid in isolation [46], the two coral snake venoms utilized in the present investigation have not been similarly tested with our methods. For example, if coral snake venoms were exposed to varespladib in isolation and then tested in our system with similar results, then it would have been confirmed that the anticoagulation observed was secondary to PLA_2_ activity. This is a significant limitation, as there is a small possibility that other enzymes in the *M. tener* and *M. fulvius* venoms could in some way unexpectedly affect coagulation beyond the effects attributable to anti-factor Xa activity. However, both *M. fulvius* and *M. tener* venoms’ anticoagulant effects in human plasma were totally abrogated by varespladib in vitro, as reported in a comprehensive manuscript by the Fry group [43]. These authors meticulously documented the Factor Xa and prothrombinase inhibition of these two venoms (Table 1, page 92) and the varespladib-mediated inhibition of both venoms (Figure 2, page 94) using a clotting-based method with recalcified, sodium-citrate-anticoagulated human plasma [43]. The return of clotting time to normal in varespladib/coral snake venom-exposed samples observed in this work [43] is analogous to the return of TMRTG and MRTG to values not different from control values in our system, as seen in Figure 3 and Figure 4. TMRTG and MRTG are the clotting time equivalents of the Fry group’s system [43]. Thus, while we again acknowledge not using varespladib in our system as a significant limitation, the basic components of the two systems are the same in terms of substrate (human plasma) and a device to determine the onset of clot formation [43]. If one considers the analogy of adding heparin to plasma to activate antithrombin to either the Fry group’s system or our thrombelastographic system, we would both be able to demonstrate anticoagulation; further, both systems would be able to demonstrate heparin neutralization by the addition of protamine to plasma as well. In summary, these are the major limitations of this investigation.

Looking beyond the present work, there may be concerns that Ru-containing compounds may adversely affect the patient or companion animal being treated. While longer-term investigations are needed and are planned, in vivo experimentation involving subcutaneous injection of a CORM-2/RuCl_3_-containing antivenom has not resulted in measurable harm based on general appearance, vital signs, behavior, or thrombelastography [59]. While it is unlikely that Ru-containing compounds only inhibit snake venom serine proteases, metalloproteinases, and PLA_2_, there are good reasons to believe that the envenomed subject may not suffer the severe inhibition of their enzymes. The mechanism of action of venomous snake bite involves injection into the interstitial space of the victim, not the intracellular spaces of the tissue’s cells. Similarly, injection of Ru-based enzyme inhibitors occurs within the same interstitial space as the snake’s fang. Thus, it is a matter of tissue compartment location, not species-specific enzyme characteristics, that determines whether venom or the victim’s enzymes are inhibited by Ru-based compounds. Besides venom, the interstitial space of the envenomed subject contains abundant proteins, such as albumin, that in turn contain numerous histidine and other amino acids known to rapidly bind Ru-containing compounds [50]. Such binding would rapidly clear the interstitial space of free Ru-containing compounds, minimizing systemic delivery. It should also be noted that Ru-based antivenom administration is dosed to inhibit venom at the bite site, using amounts and concentrations likely to inhibit the venom that are far below those needed to inflict measurable toxicity to the bitten subject, as reviewed in [59]. Thus, when considered together, it is anticipated that the administration of these Ru-based, precision enzyme inhibitors will primarily inactivate harmful venom enzymes while minimally interacting with the bite victim’s enzymes. Or, put another way, Ru-containing compounds, as administered [59], are likely far less damaging to the tissues of a snake bite victim than untreated venom.

Precision snake venom enzyme inhibitors that are site-directed are relatively new as a concept. We have found that Ru-based mixtures of compounds act as snake venom enzyme inhibitors by inhibiting multiple toxic effects in vitro [46,47,48,51,52,64,65] and critically hemotoxic venoms in vivo [59]. Medications such as varespladib are viewed as adjunctive to the administration of traditional, antibody-based antivenoms, as only one toxin is inhibited. However, macromolecules such as antivenom antibodies cannot neutralize interstitial venom toxins within and surrounding the bite site, as evidenced by re-envenomation within two weeks after treatment for rattlesnake bite [66,67,68,69,70,71,72,73,74,75,76,77,78,79,80]. Recurrence of symptoms can require administration of additional intravenous, antibody-based antivenoms [66,67,68,69,70,71,72,73,74,75,76,77,78,79,80]. Recurrent envenomation is a very serious problem, affecting patients exposed to hemotoxic or neurotoxic venoms from snakes worldwide [66,67,68,69,70,71,72,73,74,75,76,77,78,79,80]. Animals also experience the phenomenon, as seen in dogs bitten in Florida, USA [72]. However, if the venom toxins within the bite site and surrounding tissues are injected with Ru-containing compounds into the interstitial space, the resulting inactivation of these multiple classes of enzymes could prevent local tissue injury and future release and consequent systemic injury. Ru-containing compounds would likely be effective hours after envenomation, as the venom would still remain at the bite site secondary to edema, ecchymosis, and compromised lymphatic drainage. The molecular promiscuity of Ru-containing compounds for inhibition of snake venom enzymes renders the group as effective in situ enzyme inhibitors that have been translated in the case of hemotoxic venoms [59]. Direct, interstitial inactivation of multiple venom enzymes is very different from administration of agents such as varespladib. Critically, our non-physiological, in vitro findings concerning inhibition of PLA_2_-mediated anticoagulation remain to be translated into a preclinical model of neurotoxicity.

In conclusion, snake venom PLA_2_ anticoagulant activity is inhibited by RuCl_3_ in a group-dependent manner, with group IA PLA_2_ only inhibited in an NS environment and group IIA PLA_2_ inhibited in an NS or PBS environment. Future investigation is warranted to determine the precise molecular sites within snake venom PLA_2_ that are vulnerable to RuCl_3_ inhibition, as well as to determine if pH and/or variations in RuCl_3_-derived ions are responsible for the observed differential inhibition. Our findings should not be considered a guarantee that Ru-containing compounds will act as enzyme inhibitors in the presence of neurotoxic venoms despite the fact that they do so in the case of hemotoxic envenomation [59]. Rigorous, in vivo validation of Ru-based compound effects on neurotoxicity associated with either group IA or IIA remains a critical translational issue that this laboratory will address in the near future, utilizing our rabbit model of envenomation [59].

## 4. Materials and Methods

### 4.1. Plasma, Venoms, and Chemicals

Pooled normal human plasma, which was sodium-citrate-anticoagulated and maintained at −80 °C, was obtained from George King Bio-Medical (Overland Park, KS, USA). Lyophilized *M. tener*, *M. fulvius*, and *C. scutulatus* venoms were provided by the National Natural Toxins Research Center (NNTRC) located at Texas A&M University-Kingsville, Kingsville, TX, USA. The National Institutes of Health funds the NNTRC out of the Office of Research Infrastructure Programs. Venom was dissolved into 0.9% NaCl (Millipore Sigma, Saint Louis, MO, USA) to a final concentration of 50 mg/mL, aliquoted, and maintained at −80 °C. RuCl_3_ and calcium-free PBS were obtained from Millipore Sigma (Saint Louis, MO, USA). Calcium chloride (200 mM) was obtained from Haemonetics Inc. (Braintree, MA, USA).

### 4.2. Thromboelastographic Analyses

The methodology for thromboelastographic analyses can be found in detail in previous works [51,52]. In brief, sample mixtures (always summing to 360 µL) were placed in a disposable cup in a computer-controlled thrombelastograph^®^ hemostasis system (Model 5000; Haemonetics Inc., Braintree, MA, USA) at 37 °C. The mixture used in a series of experiments was composed of 320 µL of plasma; 16.4–20 µL of PBS; 3.6 µL of venom in NS; 3.6 µL of 100 µM RuCl_3_ in NS; 3.6 µL of 100 µM RuCl_3_ in PBS; 3.6 µL of 100 µM RuCl_3_ and venom in NS; 3.6 µL of 100 µM RuCl_3_ in PBS; 3.6 µL of 100 µM RuCl_3_ and venom in PBS; and 20 µL of calcium chloride. Prior to addition to plasma, the venoms were exposed to only NS or 100 µM RuCl_3_ in NS or PBS for at least 5 min at room temperature. Venom solutions were always added to the plasma mixture just prior to the addition of calcium chloride to prevent any premature, unmonitored degradation of coagulation from occurring. After calcium chloride addition and mixing by moving the cup up and down on a pin three times, data collection was performed for 25 min.

### 4.3. Statistical Analyses

Data are presented as means + SD. All conditions were represented by n = 6 replicates, as this provides a statistical power of ≥0.8 with *p* < 0.05, and this methodology was used to assess differences in thrombelastographic parameters [42,51,57,58,64,65]. A commercially available statistical program was used for one-way analysis of variance (ANOVA), followed by Holm–Sidak post hoc analyses (SigmaStat 3.1; Systat Software, Inc., San Jose, CA, USA). Graphics were generated with commercially available programs (Origen 2026, OrigenLab Corporation, Northampton, MA, USA, and CorelDRAW 2024, Alludo, Ottawa, ON, Canada). *p* < 0.05 was considered significant.

## Figures and Tables

**Table 1:** Relationships of inhibition of snake venom enzymes and Ru-containing compounds with the cited literature.

Snake Venom Enzyme	CORM-2	RuCl_3_ In NS	RuCl_3_ In PBS
Serine Protease	+ [64]	+ [52]	+/− [52,65]
Metalloproteinase	+ [64]	+ [51]	+ [51]
PLA_2_ (group IA)	+ [48]	+	-
PLA_2_ (group IIA)	+ [46]	+	+ [47]

## Data Availability

The original contributions presented in this study are included in the article. Further inquiries can be directed to the corresponding author.
